# Ontogeny of postgenital leaf sheath fusion in *Commelina erecta* L. (Commelinaceae)

**DOI:** 10.1007/s00425-026-05029-4

**Published:** 2026-05-30

**Authors:** Arthur K. Chan, Michael J. Scanlon, Gladys F. A. Melo-de-Pinna

**Affiliations:** 1 Plant Anatomy Laboratory, Institute of Biosciences, University of São Paulo, São Paulo, Brazil; 2 Plant Sciences Building, College of Agriculture and Life Sciences, Cornell University, Ithaca, USA

**Keywords:** KNOX, Commelinaceae, *Commelina erecta*, Sheath, Leaf development

## Abstract

**Main conclusion:**

* Commelina erecta* L. leaf sheaths fuse postgenitally without KN1 ortholog expression, indicating a distinct molecular mechanism for sheath fusion in *Commelinaceae*, contrasting it from sheath formation in *Poaceae*.

**Abstract:**

Leaf sheaths are common structures within monocotyledonous, being open or closed depending on the separation of their margins. In Commelinaceae, a family of monocotyledonous plants with simple leaves and alternate phyllotaxy, their sheaths are closed. In Poaceae, which often have open sheaths, there has been evidence of *KNOX* genes affecting the fusion in sheaths in this family. The present study, therefore, investigated the nature of the sheath fusion and the expression of *KN1* ortholog at the leaf sheaths in *Commelina erecta* L. Samples of the shoot apex and leaf primordia were subjected to scanning electron microscopy and optical microscopy techniques with serial sections in paraffin. Samples of apical meristems from specimens of *C. erecta* were also subjected to in situ hybridization to verify the expression of *KNOX* within its leaves, with the probe sequence established by degenerate RT-PCR. The anatomical analysis revealed that sheaths of *C. erecta* undergo post-genital fusion. In situ hybridizations in *C. erecta* revealed no expression of *KN1* ortholog at the region of the fused sheath margins*.* The post-genital fusion indicates, therefore, the previous separation of the sheath margins, which, associated with lack of expression of *KN1* at this region, suggests a distinct molecular mechanism of leaf fusion in Commelinaceae, contrasting it from sheath formation in Poaceae.

**Graphic abstract:**

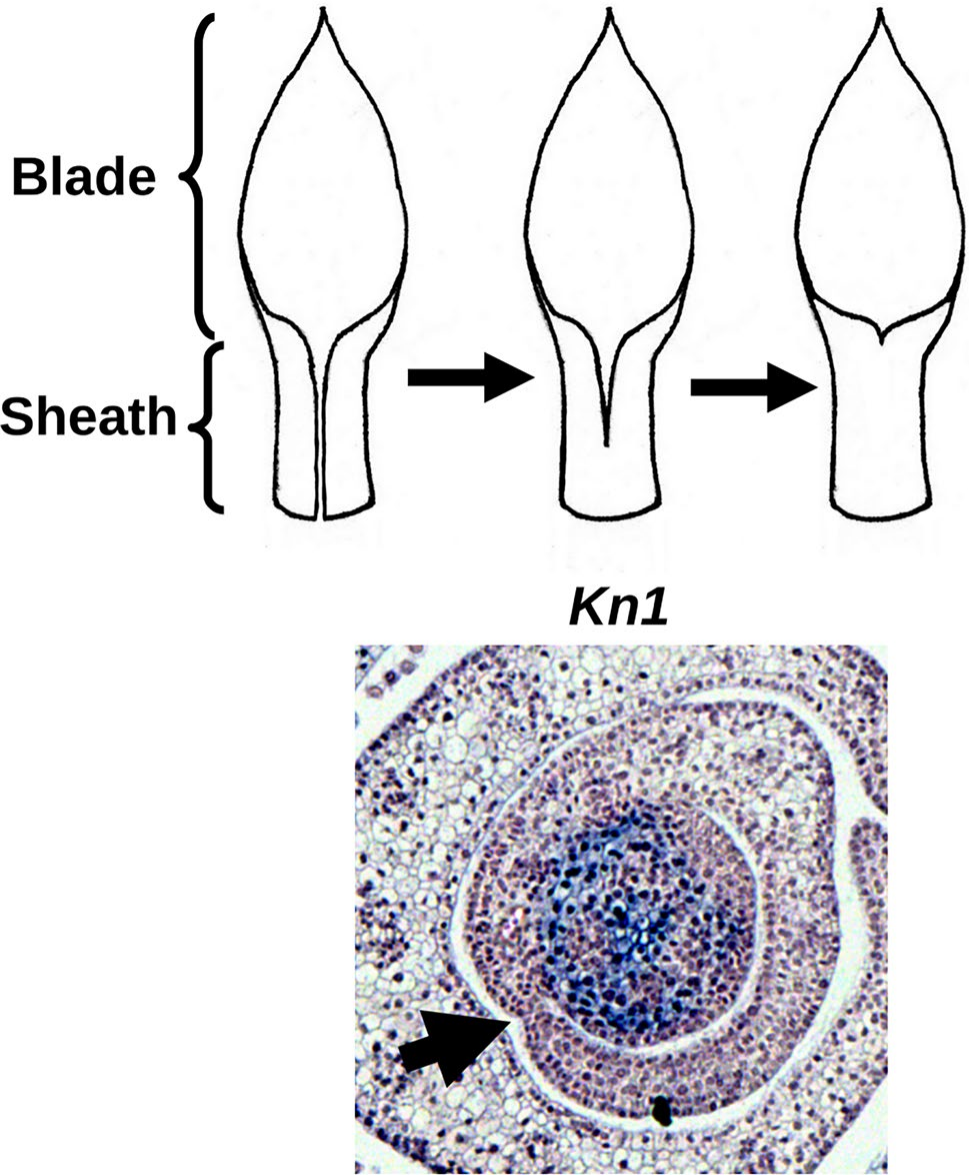

**Supplementary Information:**

The online version contains supplementary material available at 10.1007/s00425-026-05029-4.

## Introduction

Leaf development in angiosperms is complex and depends on the interactions of genetic regulators and the actions of phytohormones, such as auxin, cytokinin, and gibberellin, that modulate their morphology and anatomy (Melo-de-Pinna & Cruz 2020). In monocotyledons, the leaves are usually subdivided into a distal lamina and a basal sheath, a structure that protects the shoot apical meristem (SAM) (Esau 1965; Faden 1998). The leaf sheath has been the subject of phylogenetic and ontogenetic studies in recent years focusing on the molecular development of its margins (Richardson & Hake 2018; Richardson et al. 2021b).

Regarding this theme, a notable case can be found in Commelinaceae, a family of herbaceous and perennial monocotyledonous plants, sometimes epiphytic, described as having simple leaves with smooth margins, alternate distichous or spiral phyllotaxy, an absence of ligules, often involute vernation and closed tubular sheaths (Faden 1998). Many species of Commelinaceae present in the leaf sheaths a line of trichomes parallel to the stem axis, occurring in the region opposite to the leaf blade, sometimes extending to their respective internodes (Saunders 1922). This paper postulated that the line of hairs located in the sheath of these species is signaling the region of the fused margins, thus being a likely marker of the marginal domain in this region.

Fusions in plant organs can be postgenital, occurring after an initial separation of the organs, or congenital, never having had a previous separation between them; in some plants postgenital fusion predominates in the apical positions of the plant, whereas congenital fusion prevails in the basal portion (Verbeke 1992; Sokoloff et al. 2018). However, it is still unknown what type of fusion occurs in the sheaths of Commelinaceae.

The closed sheath morphology in the Commelinaceae contrasts with those found in Poaceae, which generally develop an open sheath whose margins typically overlap, but exhibit no fusion (Esau 1965; Dahlgren et al. 1985; Richardson & Hake 2018). The leaf primordia of Poaceae are ring-shaped, in such a way that they are inserted into the stem axis by a circular base called the “disc of insertion” (Sharman 1942). The delimitation of sheath margins is, therefore, necessary for their future separation to occur. It is known that in *Zea mays* the delimitation of these regions depends on the transport of auxin, as detected by the expression of *SPARSE INFLORESCENCE1* (*SPI1*), and auxin biosynthetic gene that is expressed at the edge of the two future margins (Johnston et al., 2015; Richardson & Hake 2018).

The influence of this auxin on the leaf sheath was also tested by applying an auxin inhibitor to *Zea mays* L. individuals grown in growth medium (Scanlon 2003). Under these conditions, the sheath of the fourth plastochron was unable to separate its margins and developed into a tubular shape, presenting a comparable morphology as the fused leaf sheaths of Commelinaceae.

Application of an auxin inhibitor in *Zea mays* also resulted in the ectopic accumulation of *KN1*, belonging to the KNOX class 1 gene family, in the closed sheaths, precisely in the region where the two margins would be defined (Scanlon 2003). Class 1 KNOX proteins promote indeterminacy in the SAM and expanding internodes, but are down-regulated in newly initiated leaf primordia (reviewed in Tsuda & Hake 2016). The ectopic presence of *KN1* in the overlapping sheaths of *Zea mays*, on the other hand, raised the hypothesis that they are also responsible in this species for the determination and separation of the left and right margins (Scanlon 2003). This data raises questions as to the organ-specific expression of *KN1* orthologs in Commelinaceae, specifically whether or not there is *KNOX* accumulation at the site of sheath margin fusion. Assuming that the molecular regulatory system remains conserved between Poaceae and Commelinaceae, the regulation of leaf primordia development by these genes could bring a better understanding of the developmental process of the fusion of their sheath margins in Commelinaceae, in addition to the role of *KNOX* ortholog in this process. However, it is still not known how this gene are expressed in the meristems of Commelinaceae; investigations are complicated by the lack of a sequenced genome.

Thus, the present study combines anatomical analyses with cell/tissue-specific investigations of gene expression during the development of closed sheath margins within *Commelina erecta* L.

## Materials and methods

### Scanning electron microscopy (SEM)

The formation of the sheath margins of *Commelina erecta* L. was investigated by Scanning Electron Microscopy (SEM). Samples of leaf primordia of *Commelina erecta* L. were subjected to dehydration in an increasing alcoholic series (70% ethanol, 85% ethanol, 90% ethanol, and twice in 100% ethanol) at 2 h intervals. They were then dried at the critical point of CO_2_, mounted on stubs and covered with gold for 2 min and subsequently examined under SEM (Silveira 1989). The observation was made using a Zeiss Microscope (DSM 940), belonging to the Electron Microscopy Laboratory of the Institute of Biosciences of the University of São Paulo.

### Light microscopy

Samples of vegetative shoot apices of 3–5 individuals of each species were fixed in FAA 50% (formalin, ethanol 50% and acetic acid) subjected to an ascending butanol series, then transferred to mineral oil and embedded in Paraplast Plus^®^, following the methodology described by Johansen (1940). The samples were cross-sectioned at a thickness of 10 μm, in a rotary microtome, subsequently deparaffinized and stained with Astra Blue 1% and Safranin 50%, and then mounted on permanent slides in Entellan^™^ (Ruzin 1999).

The photographic record was made using the IM50 Image Digitization System, coupled to the Leica DMLB microscope of the Plant Anatomy Laboratory of the Institute of Biosciences of the University of São Paulo.

### Sample preparation for in situ hybridization (Strable & Satterlee 2021)

Samples of apical meristems from *Commelina erecta* L. individuals were subjected to in situ hybridization to verify the expression and anatomical position of orthologs of *KN1* in the sheaths of Commelinaceae (Strable & Satterlee 2021).

The stem tips of *C. erecta* were cut and fixed in FAA 3.7% (Formalin, Ethanol 3.7%, Acetic Acid) with the vials in a container with ice, followed by an ethanol series at 4ºC, under gentle agitation, and left overnight. The material was transferred to Paraplast Plus^®^ inside an oven at 60ºC, where it remained for 3 days, with the paraplast being renewed twice a day.

The day before in situ hybridization, the incorporated material of *C. erecta* was sectioned with 10 µm slices following the methodology of Ruzin (1999), with the paraplast tapes extended on Probe-On-Plus slides with the application of water treated with DEPC (diethyl dicarbonate).

### Gene cloning and probe preparation

RNA from *C. erecta* was extracted and subsequently reverse-transcribed into complementary DNA (cDNA). Fragments of *KN1* homologue were then isolated by polymerase chain reaction (PCR). Due to the lack of sequenced genomes in Commelinaceae, the probe sequence was established by degenerate RT-PCR at an alignment temperature of 40 °C, based on conserved regions of these genes in species closest to *Commelina* with sequenced genomes (Ji et al 2010), with members of *Musa* and *Zingiber* (Order Zingiberales) being the closest sequences available. All reactions were prepared with 1 μl of cDNA and 9 μl of master mix and 1.5 μl for each primer diluted 1/10 (adding 3 μl for the forward and reverse primers), totaling 18 μl for the entire reaction. The designed primers can be found in Table 1.

**Table 1:** Nucleotide sequences of the designed primers of *KN1*, with positions containing variable nucleotides marked with the IUPAC degenerate nucleotide code

Primer	Sequences
*KN1* FWD	GGTSAARTWCAGGGARGAGC
*KN1* REV	CKCKTCCKYTGRTTKATGAACC

### Phylogenetic analysis

The acquired sequences from the probe of the ortholog of *KN1* of *C. erecta* (available at Online Resource 1) had its protein sequence translated, and was compared with protein sequences recorded from members of the *KNOX* gene family, obtained through BLASTN searches in NCBI (Table 2). These sequences were then aligned by the ClustalW1.4 algorithm, and visualized through the Bioedit software (Hall 1999). The same sequences were then analyzed phylogenetically through the maximum likelihood criterion, with the selection of models and phylogenetic analyses performed through the IQTREE program (Trifinopoulos et al. 2016), and visualized with iTol software (Letunic and Bork 2024).

**Table 2:** *KN1* orthologs used for the alignment and phylogenetic analysis

Ortholog	Species	Family	Order	Genbank accession number
AaKN1	*Asparagus asparagoides*	Asparagaceae	Asparagales	BAM08928.1
AtKNAT1	*Arabidopsis thaliana*	Brassicaceae	Brassicales	AAM45030.1
CpKN1	*Cucurbita pepo*	Cucurbitaceae	Cucurbitales	XP_023536402.1
BpKNOX3	*Brachypodium distachyon*	Poaceae	Poales	XP_010230586.1
OsKN1	*Oryza sativa*	Poaceae	Poales	NP_001389174.1
OsOSH1	*Oryza sativa*	Poaceae	Poales	ABF98653.1
PhKN1	*Panicum hallii*	Poaceae	Poales	XP_025795239.1
TaKN1	*Triticum aestivum*	Poaceae	Poales	XP_044368246.1
ZmKN1	*Zea mays*	Poaceae	Poales	ONM08817.1
TlKN1	*Typha latifolia*	Typhaceae	Poales	XP_072989408.1
CiKN1	*Canna indica*	Cannaceae	Zingiberales	WOK92258.1
MaKN1	*Musa acuminata*	Musaceae	Zingiberales	XP_009411331.3
ZoKN1	*Zingiber officinale*	Zingiberaceae	Zingiberales	XP_042444176.1

### In situ hybridization

The procedure for In situ hybridization is based on Jackson (1992) and Juarez et al. (2004), described below: the slides transported to a metal support were deparaffinized in histoclear solution, followed by a descending ethanol series, application of PBS (NaCl and Na_2_HPO_4_) and an ascending ethanol series. The material was then stored in an airtight container with a little ethanol at the bottom.

After preparation, the *KN1* probe was then diluted, denatured at 80 °C, cooled on ice, and briefly centrifuged. The processed probe was then added to the hybridization solution (NaCl, Tris–HCl, sodium phosphate, EDTA, formamide, dextran sulfate, Denhardt's solution, and nonspecific tRNA), and the mixture was spread on the slides. The slides were then adhered in pairs and stored inside a box with formamide, remaining overnight at 50 °C. The next day, the slides were subjected to a series of washes with NTE buffer, saline-sodium citrate buffer (SSC), Roche Blocking Solution, and BSA/Triton-X 100 solution, when the anti-digoxigenin antibody was subsequently added to the material. After two hours, the slides were washed with TBS and TN buffers and left overnight. Once the staining was sufficiently clear, the slides were washed with TE (Tris–HCl and EDTA), rapidly dehydrated with an ethanol series, washed in histoclear, dried and mounted with Permount. The images were analyzed and taken with a Leica DMLB optical microscope.

## Results

### Sheath formation

**Fig. 1:**
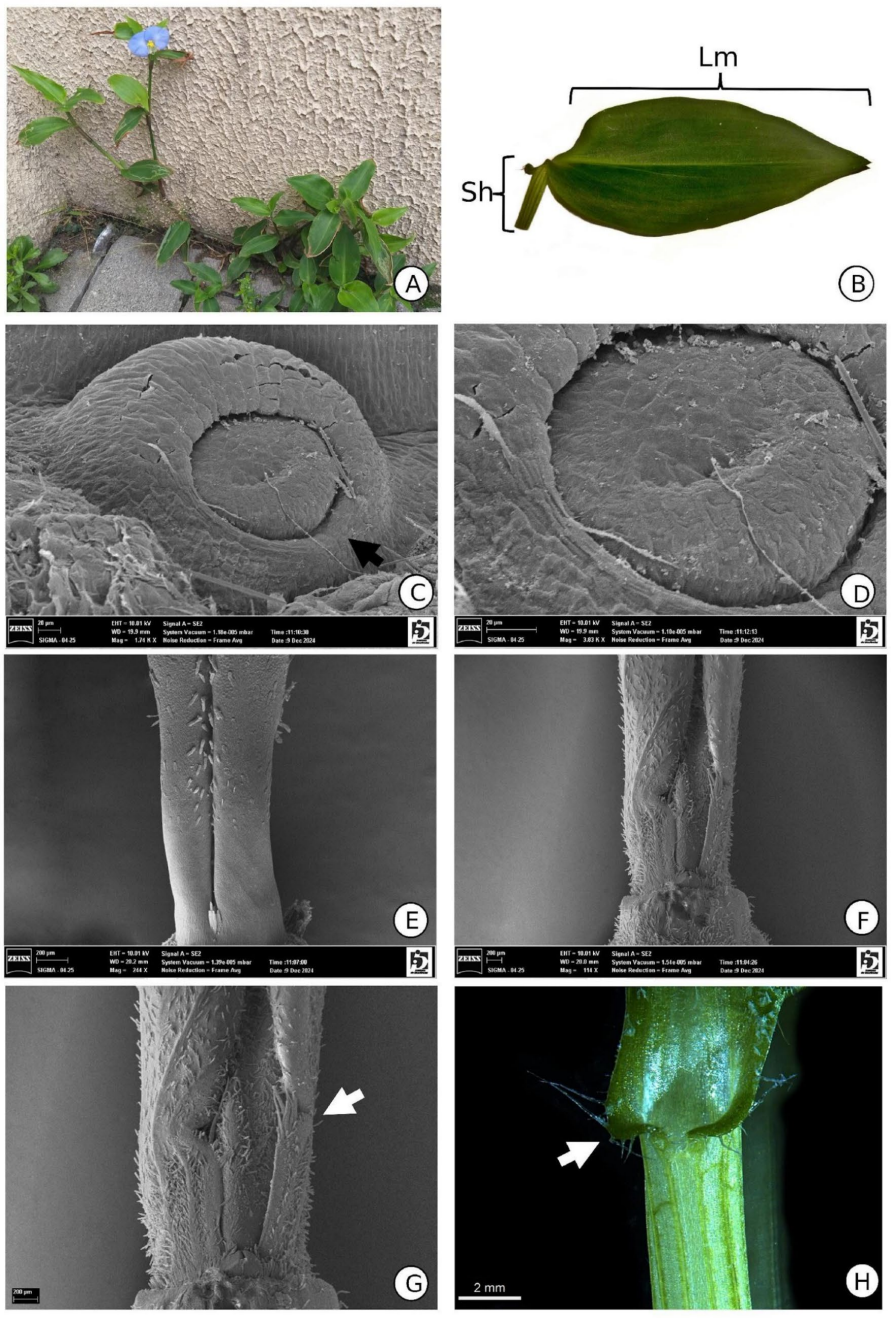
Stages of development of the *Commelina erecta* sheath. **A** Adult plant. **B** An overview of the adult leaf. Lm – Limb. Sh – sheath. **C–D**. Initiation and primary morphogenesis, with the region of the constitutive base marked by the arrow. **E** Stage of expansion of the leaf blade, still without the expanded sheath. F-G. Stage of expansion of the sheath, with free margins; with the upper portion of the sheath delimited by the presence of the region of formation of the future auricle, marked by the arrow. H. Adult leaf, with the presence of the closed sheath and auricles (arrow)

**Fig. 2:**
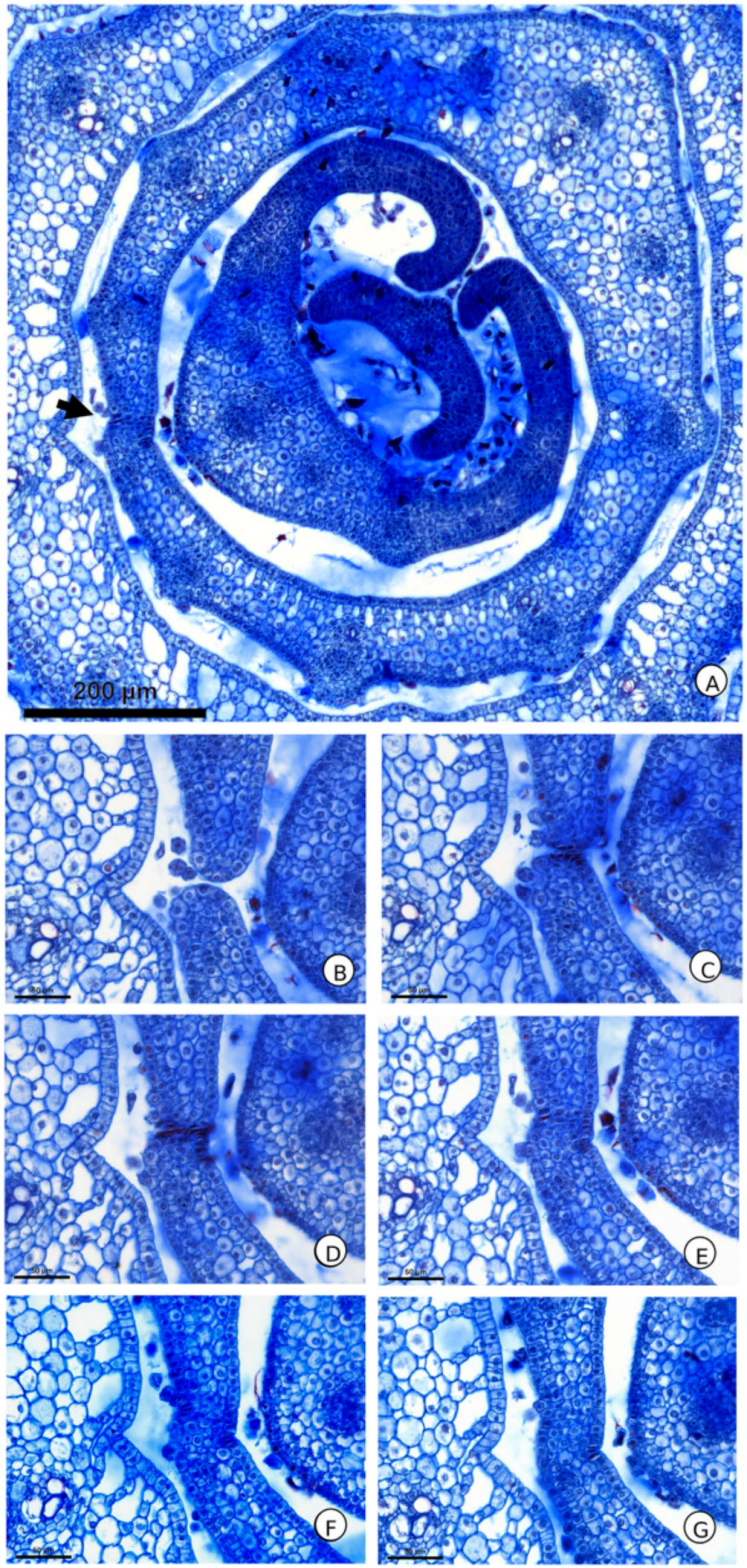
*Commelina erecta* sheath in primordium at the fifth plastochron, undergoing the process of postgenital fusion revealed in serial transverse sections of the stem apical region. **A** Region of fused margins marked by the arrow. **B** Region of the sheath with free margins. **C**–**E** Transition between free and fused portion of the sheath. **F**–**G**. Region with fused margins

During later stages of the leaf primordia in *C. erecta*, the sheaths develop free margins (Fig. 1), around the fourth to fifth plastochron. This is evident by the fact that the margins of the sheath are demarcated from the leaf blade by the presence of clefts delimiting the future auricles, characteristic of the species. Earlier stages of leaf primordia development, from the third to fourth plastochron, show a little expansion of the sheath, but pronounced growth of the leaf blade and internode, revealing that the sheath expands later in relation to these regions (Fig. 1). Cross sections at the apex of the fused portion of the sheath of *C. erecta* (Fig. 2) reveal a histological transition of the epidermis of the two margins in contact: initially separated, the two epidermis layers come into contact, undergo cellular changes as indicated by the presence of elongated, intervening cells that will eventually become part of the mesophyll.

### Gene orthology

Candidate *C. erecta* ortholog of *KN1*, *CeKN1*, was isolated using degenerate PCR (Table 1). Alignments of the *C. erecta KN1* ortholog with the corresponding sequenced genes (available, respectively, at Online Resources 2 and 3) revealed the presence of conserved regions in all genes, thus indicating the phylogenetic proximity of the obtained sequences with the respective gene families.

**Fig. 3:**
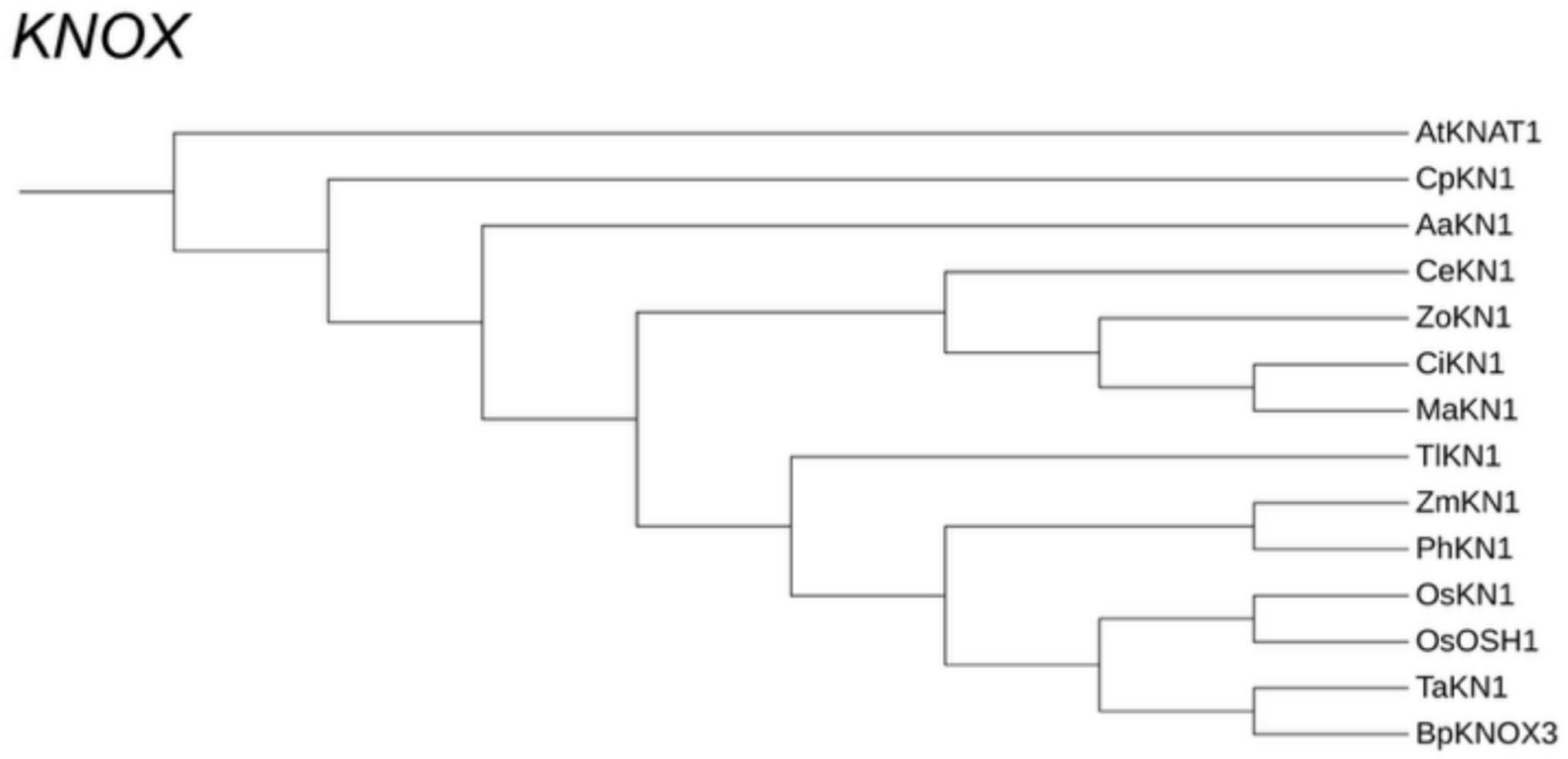
Phylogenetic *KNOX* tree obtained through maximum likelihood analysis of protein sequences obtained from *Commelina erecta* with the cl osest sequences available. Root positioned in *Arabidopsis thaliana KNAT1*

Phylogenetic likelihood analyses showed that the *CeKN1* ortholog is phylogenetically close to the other genes (most significantly *KN1*) within the *KNOX* Class 1 family in a selected range of angiosperms (Fig. 3A). In particular, significant sequence conservation is found in *KN1* orthologs from the Zingiberales (*Musa acuminata* and *Zingiber officinalis*). These findings are in phylogenetic agreement, since Zingiberales is the sister group of Commelinales, which includes *C. erecta*.

### In situ hybridization of* KN1* homolog in* C. erecta*

**Fig. 4:**
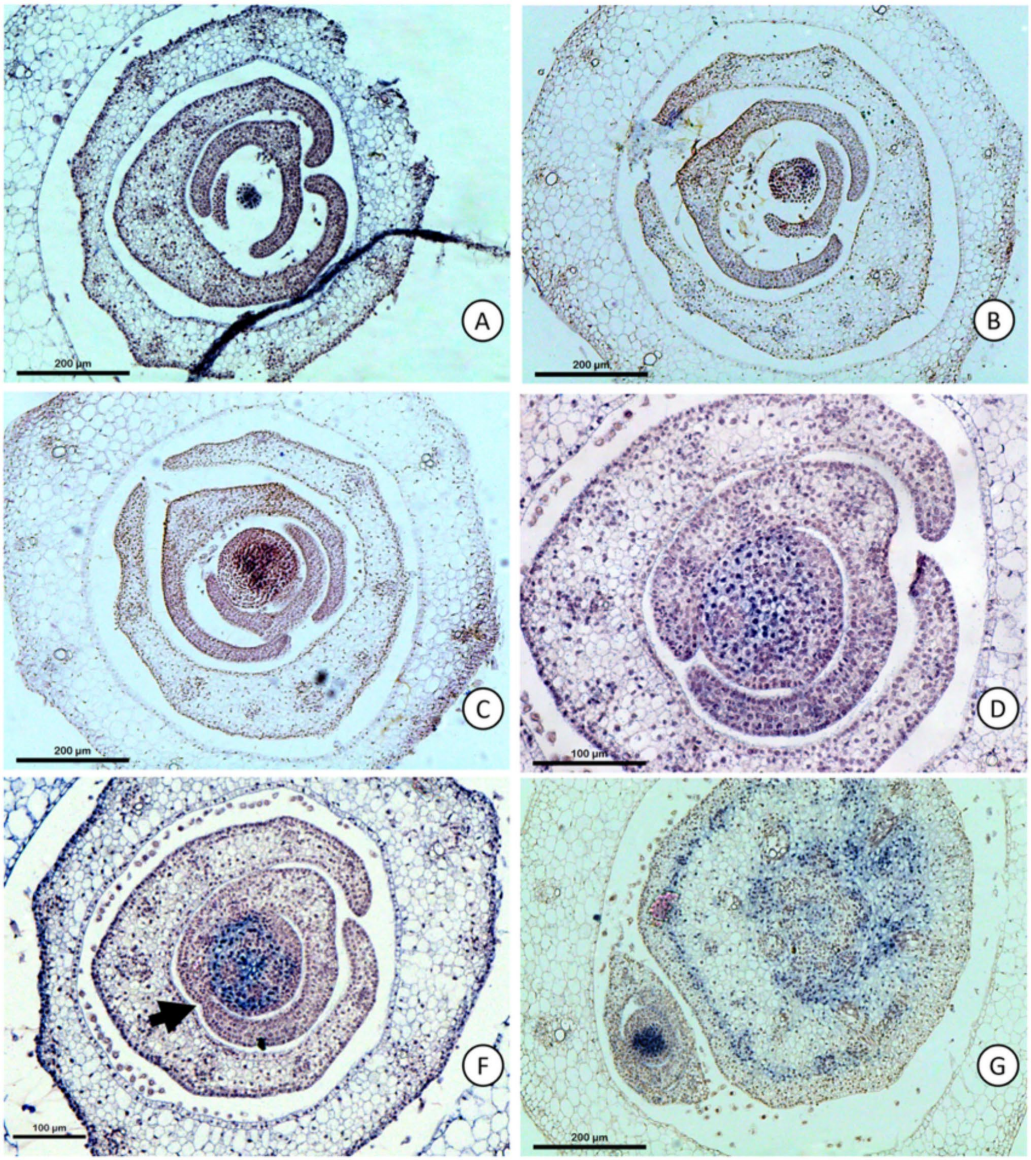
In situ hybridization of the *KN1* ortholog of *C. erecta*, observed in transverse sections of the SAM. Region of the fused margins of the sheath marked with an arrow

Transverse sections of *C. erecta* do not reveal accumulation of *CeKN1* transcripts in the region of the fused margins of the sheath nor in other portions of the leaf primordium. In contrast, *CeKN1* expression was detected only in the apical and axillary meristems extending to the first internodes (Fig. 4), but with a clear absence of transcript accumulation in the region of the leaf primordial disc of insertion.

On the other hand, *CeKN1* was expressed along the stem axis as a peripheral ring in the cortex region. This ring can be observed up to the fourth internode region. Right in the region of the leaf insertion disc, this ring is found exactly below the forming larks. *CeKN1* is also strongly expressed in the stem pith, in procambium cells.

**Fig. 5:**
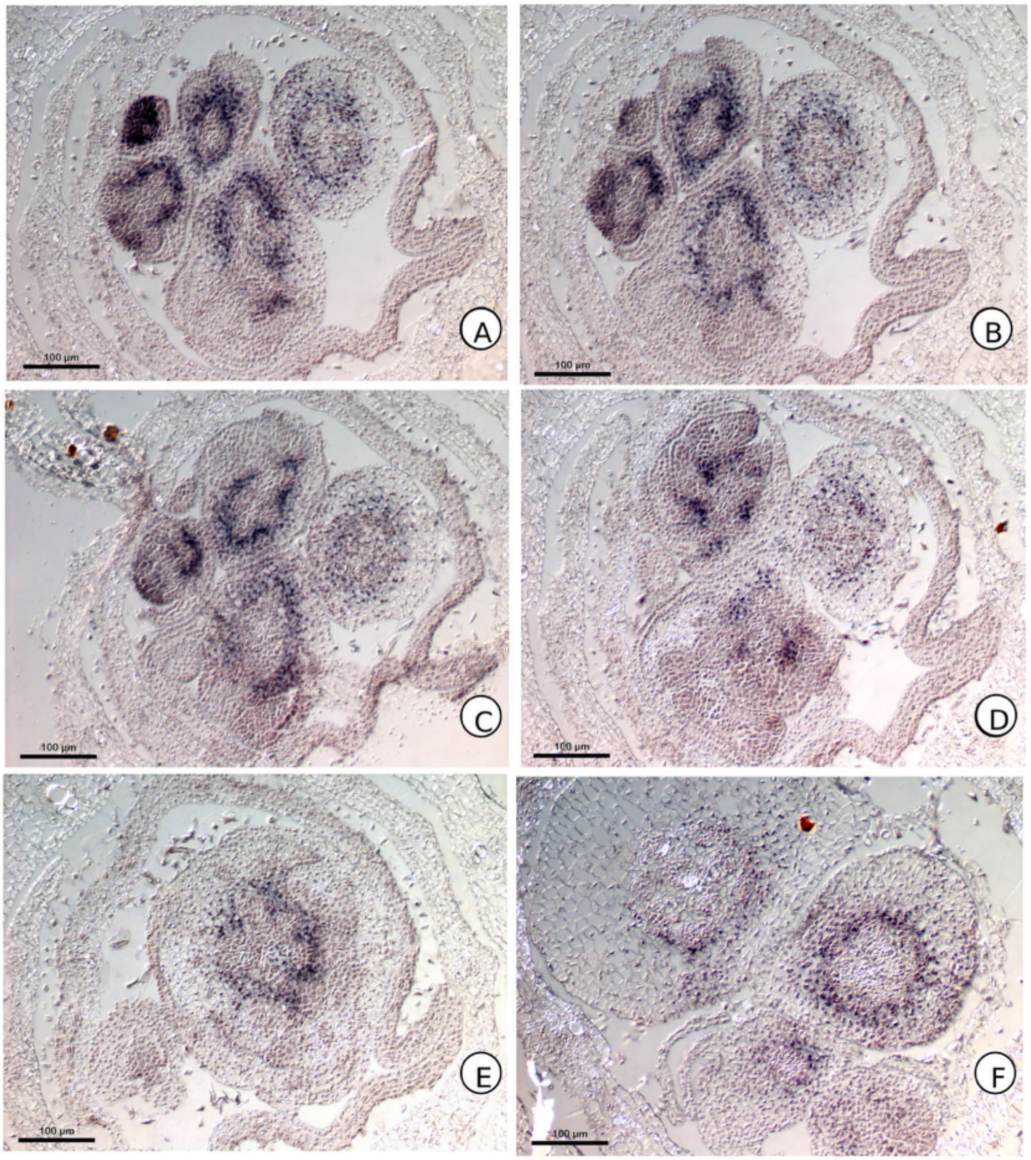
In situ hybridization of the *C. erecta KN1* ortholog, observed in transverse serial sections of the inflorescence, revealing the expression of *KN1* at the floral meristems and developing vascular system

In situ hybridization images also reveal that *CeKN1* is also expressed in floral meristems, in addition to being present in the central region of the developing vascular system (Fig. 5).

## Discussion

### Leaf sheathes in *Commelina erecta* develop by postgenital fusion

The presence of leaf primordia in which the two margins of the sheath are separated (Fig. 1) indicates a process of postgenital fusion in Commelinaceae. This reveals that there was redifferentiation of the cells in the region where the margins of the sheath overlap into parenchyma cells, which are supported by the presence of histological changes in the epidermal cells at the site of fusion (Fig. 2). The existence of postgenital fusion, therefore, implies the existence of the process of margin separation, which delimit the region of the marginal domain of the leaf.

### *CeKN1* expression delimits vascular and meristematic domains and reveals distinct mechanisms of sheath fusion in *Commelina erecta*

Class 1 KNOX genes accumulate in the SAM, reproductive meristems and non-elongating internodes, but generally not in developing leaf primordia, where they are down-regulated. They are responsible for meristem indeterminacy and maintenance (Tsuda & Hake 2016). In fact, overexpression of these genes in a variety of angiosperm species examined can result in the formation of new adventitious meristems, for example, in tobacco leaves (Srinivasan et al. 2011). In this sense, the absence of ectopic accumulation of *CeKN1* in the fused margins of leaf sheaths in *C. erecta* is expected regarding the usual expression of the *KNOX* family. Furthermore, the accumulation of *KN1* in *C. erecta* is extremely similar to that obtained in results from members of Poaceae such as *Zea mays*, occurring in the SAM and growing stem, but absent in leaf primordial founder cells at the SAM periphery (Smith et al. 1992; Jackson et al., 1994). In reproductive stages, *ZmKN1* accumulates in floral meristems and in the procambium of developing pedicels. This accumulation pattern, together with the phylogenetic analysis of the probe sequence (Fig. 1), indicate that our *CeKN1* gene candidate does indeed comprise a *KNOX* ortholog.

The fact that the *CeKN1* ortholog accumulates in the procambium regions can be assessed by comparing the images obtained with the expression already described for the organization of the vascular system in Commelinaceae (Vita et al. 2019). While the peripheral ring of *CeKN1* in the cortex region (Fig. 2) corresponds to the region where the stem bundles are formed in the internodal region and the external vascular plexuses in the node region, delimited by the pericycle, the marking of the central portion corresponds to the region of the central bundles. These results indicate that the native accumulation of *CeKN1* may be delimiting the regions of the vascular system, which could reveal the role of this gene in the formation of this system. This marked accumulation of *CeKN1* transcripts in vascular tissue and its role in the formation of this system are also consistent with the expression pattern of other *KNOX* family genes, such as *STM*, *KNAT1* and *POTH1*, which are known to mark procambial regions in several species of monocotyledons and eudicotyledons (Lincoln et al. 1994; Rosin et al. 2003; Tioni et al. 2003). *KNOX* genes are also known to affect the development of the vascular system, as reported through mutant experiments, such as *KN1* in *Zea mays* (Poaceae) leaves and *KNAT1* in *Arabidopsis thaliana* (Brassicaceae) inflorescences (Veit et al. 1990; Woerlen et al. 2017). Future analyses in other members of Commelinaceae may clarify whether this pattern is conserved for other members of the family, which may bring further insight regarding the role of *KNOX* genes in vascular development.

Considering the association between *KNOX* gene expression and the vascular system, the presence in *C. erecta* of a continuous central cylinder of *CeKN1* accumulation, with an absence of expression at internal region, is consistent with the interpretations of stele by Tomescu (2021) that argues that, in Tracheophytes, there is a duality in the concept of stele, being defined both by the provascular region by the presence of endoderm, and by the procambium. According to this perspective, even medullary parenchymatic regions present in stems have a vascular origin. Thus, the expression of *CeKN1* also corroborates this interpretation of the stele.

Although *KNOX* genes are mostly known to act in meristem indeterminacy, homologues of Class 1 *KNOX* genes are implicated in sheath development in grasses and can also affect the shape of leaf margins and, in some eudicots, the formation of compound leaves (Satterlee et al. 2020; Hareven et al. 1996; Janssen et al. 1998; Bharathan et al. 2002). For example, in compound leaves of *Cardamine hirsuta, KNOX* transcripts accumulation occurs in leaf primordia, whereas in *Solanum lycopersicum* ectopic expression of *KNOX* genes can lead to the subdivision of super-compound leaflets, determining the formation of simple or compound leaves (Haraven et al. 1996; Hay & Tsiantis 2006).

In the current model for sheath development in the Poaceae, it is assumed that *KN1* orthologs are correlated with the separation of the lower margins of the sheath, as evidenced by the ectopic expression of *KN1* within the fused margins of limbs affected by auxin transport inhibitors (Scanlon 2003). On the other hand, the results of *C. erecta* show that fused sheaths can develop completely closed even in the absence of *KN1* accumulations in the fused region of their margins. Furthermore, the fact that the development of fused sheath margins in this species occurs through postgenital fusion (Fig. 1) reveals the occurrence of its margin separation in *C. erecta*. Thus, postgenital fusion associated with the absence of *KN1* in the sheath indicates a distinct molecular mechanism of fusion in *C. erecta* from that responsible for the formation of fused sheaths in *Zea mays* treated under auxin transport inhibition. Instead, postgenital fusions are known to be determined by genes such as *CRINKLY4* in *Zea mays* leaves, and *FIDDLEHEAD* in the leaves and flowers of *Arabidopsis thaliana*. These genes are involved in redifferentiation of the epidermis from the fusion regions of developing organs or in changing the permeability of the inter-epidermal contact (Lolle et al. 1998; Sieber et al. 2000; Phillips et al. 2020). However, it is currently unknown which genes are responsible for the postgenital fusion of the leaf sheaths in Commelinaceae. A transcriptome analysis of sheath regions at different stages of fusion may shed light on the mechanisms regulating postgenital fusion in this family.

However, the details of how *KNOX* genes are actually involved in the process of opening the Commelinaceae sheath margins and their relationship with auxins remain open due to the possibility of the action of other members of the gene class other than *CeKN1*. Furthermore, a comparison of the regulatory systems of *KNOX* and auxins between *Z. mays* and *S. lycopersicum* regarding the formation of leaf margins reveals changes in the regulation between *KNOX* and auxin signaling proteins, being specific even within clades (Wang et al. 2005; Richardson et al. 2021a), thus indicating the possibility that this relationship is not conserved between Poaceae and Commelinaceae, which belong to distinct orders. Future investigations are needed to clarify exactly how *KNOX* gene regulation occurs in Commelinaceae.

## Supplementary Information

Below is the link to the electronic supplementary material.

Supplementary file1 (PDF 1367 KB)

Supplementary file2 (XLS 7 KB)

## Data Availability

The nucleotides sequence data were deposited into the GenBank database under accession number SUB15983372.

## References

[CR1] Bharathan G, Goliber TE, Moore C, Kessler S, Pham T, Sinha NR (2002) Homologies in leaf form inferred from KNOXI gene expression during development. Science 296:1858–1860 12052958 10.1126/science.1070343

[CR2] Dahlgren RMT, Clifford HT, Yeo PF (1985) The families of the monocotyledons: structure, evolution and taxonomy. Springer, Berlin

[CR3] Esau K (1965) Plant anatomy, 2nd edn. John Wiley & Sons, New York

[CR4] Faden RB (1998) Commelinaceae. In: Kubitzki K (ed) Flowering plants: monocotyledons. The families and genera of vascular plants, vol 4. Springer, Berlin, pp 109–128

[CR5] Hall TA (1999) BioEdit: a user-friendly biological sequence alignment editor and analysis program for Windows 95/98/NT. Nucleic Acids Symp Ser 41:95–98

[CR6] Hareven D, Gutfinger T, Parnis A, Eshed Y, Lifschitz E (1996) The making of a compound leaf: genetic manipulation of leaf architecture in tomato. Cell 84:735–744 8625411 10.1016/s0092-8674(00)81051-x

[CR7] Hay A, Tsiantis M (2006) The genetic basis for differences in leaf form between *Arabidopsis thaliana* and its wild relative *Cardamine hirsuta*. Nat Genet 38:942–947 16823378 10.1038/ng1835

[CR9] Jackson D (1992) *In situ* hybridization in plants. In: Gurr SJ, McPherson MJ, Bowles DJ (eds) Molecular plant pathology: a practical approach. Oxford University Press, Oxford, pp 163–174

[CR10] Janssen BJ, Williams A, Chen JJ, Mathern J, Hake S, Sinha N (1998) Isolation and characterization of two knotted-like homeobox genes from tomato. Plant Mol Biol 36:417–425 9484482 10.1023/a:1005925508579

[CR11] Ji J, Strable J, Shimizu R, Koenig D, Sinha N, Scanlon MJ (2010) WOX4 promotes procambial development. Plant Physiol 152:1346–1356 20044450 10.1104/pp.109.149641 PMC2832261

[CR12] Johansen DA (1940) Plant microtechnique. McGraw-Hill, New York

[CR45] Johnston R, Leiboff S, Scanlon MJ (2015) Ontogeny of the sheathing leaf base in maize (*Zea mays*). New Phytologist 205:306–15. 10.1111/nph.13010 25195692 10.1111/nph.13010

[CR13] Juarez MT, Kui JS, Thomas J, Heller BA, Timmermans MC (2004) MicroRNA-mediated repression of rolled leaf1 specifies maize leaf polarity. Nature 428:84–88 14999285 10.1038/nature02363

[CR15] Letunic I, Bork P (2024) Interactive Tree of Life (iTOL) v6: recent updates to the phylogenetic tree display and annotation tool. Nucleic Acids Res 52:W78–W82. 10.1093/nar/gkae268 38613393 10.1093/nar/gkae268 PMC11223838

[CR16] Lincoln C, Long J, Yamaguchi J, Serikawa K, Hake S (1994) A knotted1-like homeobox gene in *Arabidopsis* is expressed in the vegetative meristem and dramatically alters leaf morphology when overexpressed in transgenic plants. Plant Cell 6:1859–1876 7866029 10.1105/tpc.6.12.1859 PMC160567

[CR17] Lolle SJ, Su WH, Pruitt RE (1998) Genetic analysis of organ fusion in *Arabidopsis thaliana*. Genetics 149:607–619 9611177 10.1093/genetics/149.2.607 PMC1460157

[CR18] Melo-de-Pinna GFA, Cruz R (2020) Leaf development in vascular plants. In: Demarco D (ed) Plant ontogeny. Nova Science Publishers

[CR19] Phillips HR, Landis JB, Specht CD (2020) Revisiting floral fusion: the evolution and molecular basis of a developmental innovation. J Exp Bot 71:3390–3404. 10.1093/jxb/eraa125 32152629 10.1093/jxb/eraa125

[CR20] Richardson AE, Hake S (2018) Drawing a line: grasses and boundaries. Plants 8:25. 10.3390/plants8010025 10.3390/plants8010004 PMC6359313 30585196

[CR22] Richardson AE, Sluis A, Hake S (2021a) The Hoja loca1 mutant and AUX/IAA function in phytomer development. bioRxiv. 10.1101/2020.03.27.01221134545362

[CR23] Richardson AE, Cheng J, Johnston R, Kennaway R, Conlon BR, Rebocho AB, Kong H, Scanlon MJ, Hake S, Coen E (2021b) Evolution of the grass leaf by primordium extension and petiole–lamina remodeling. Plant Sci 374:1377–1381 10.1126/science.abf9407 34882477

[CR24] Rosin FM, Hart SJ, Horner JL, Davies JL, Moreau SA, Sinha NR (2003) Overexpression of a knotted-like homeobox gene of potato alters vegetative development by decreasing gibberellin accumulation. Plant Physiol 132:106–117 12746517 10.1104/pp.102.015560 PMC166957

[CR25] Ruzin SE (1999) Plant microtechnique and microscopy. Oxford University Press, Oxford

[CR26] Satterlee JW, Scanlon MJ (2019) Coordination of leaf development across developmental axes. Plants 8:433. 10.3390/plants8120433 31652517 10.3390/plants8100433 PMC6843618

[CR27] Satterlee JW, Strable J, Scanlon MJ (2020) Plant stem-cell organization and differentiation at single-cell resolution. Proc Natl Acad Sci U S A 117:33689–33699. 10.1073/pnas.2018788117 33318187 10.1073/pnas.2018788117 PMC7776995

[CR28] Saunders ER (1922) The leaf-skin theory of the stem: a consideration of certain anatomico-physiological relations in the spermophyte shoot. Ann Bot 36:405–425

[CR29] Scanlon MJ (2003) The polar auxin transport inhibitor N-1-naphthylphthalamic acid disrupts leaf initiation, KNOX protein regulation, and formation of leaf margins in maize. Plant Physiol 133:597–605 14500790 10.1104/pp.103.026880 PMC219036

[CR47] Sharman BC (1942) Developmental anatomy of the shoot of *Zea mays L*. Ann Bot 6:245–282. 10.1093/oxfordjournals.aob.a08840710.1093/oxfordjournals.aob.a088407

[CR30] Sieber P, Schorderet J, Ryser J, Buchala JP, Kolattukudy P, Métraux JP, Nawrath E (2000) Transgenic *Arabidopsis* plants expressing a fungal cutinase show alterations in the structure and properties of the cuticle and postgenital organ fusions. Plant Cell 12:721–737 10810146 10.1105/tpc.12.5.721 PMC139923

[CR31] Silveira M (1989) Preparação de amostras biológicas para microscopia eletrônica de varredura. Manual sobre técnicas básicas em microscopia eletrônica. Universidade de São Paulo, São Paulo, pp 71–79

[CR32] Smith LG, Greene B, Veit B, Hake S (1992) A dominant mutation in the maize homeobox gene *Knotted-1* causes its ectopic expression in leaf cells with altered fates. Development 116:21–30. 10.1242/dev.116.1.21 1362381 10.1242/dev.116.1.21

[CR33] Sokoloff DD, Remizowa MV, Timonin AC, Oskolski AA, Nuraliev MS (2018) Types of organ fusion in angiosperm flowers (with examples from Chloranthaceae, Araliaceae and monocots). Biol Serb 40:16–46

[CR34] Srinivasan C, Liu Z, Scorza R (2011) Ectopic expression of class 1 KNOX genes induces adventitious shoot regeneration and alters growth and development of tobacco (*Nicotiana tabacum* L.) and European plum (*Prunus domestica* L.). Plant Cell Rep 30:655–664. 10.1007/s00299-010-0993-7 21212958 10.1007/s00299-010-0993-7

[CR35] Strable J, Satterlee JW (2021) Detecting spatiotemporal transcript accumulation in maize by RNA *in situ* hybridization. Bio Protoc 11:e3924. 10.21769/BioProtoc.3924

[CR36] Tioni MF, Gonzalez DH, Chan RL (2003) Knotted1-like genes are strongly expressed in differentiated cell types in sunflower. J Exp Bot 54:681–690 12554711 10.1093/jxb/erg077

[CR37] Tomescu AMF (2021) The stele – a developmental perspective on the diversity and evolution of primary vascular architecture. Biol Rev 96:2025–2051 10.1111/brv.12699 33655608

[CR38] Trifinopoulos J, Nguyen LT, von Haeseler A, Minh BQ (2016) W-IQ-TREE: a fast online phylogenetic tool for maximum likelihood analysis. Nucleic Acids Res 44:W232–W235. 10.1093/nar/gkw256 27084950 10.1093/nar/gkw256 PMC4987875

[CR39] Tsuda K, Hake S (2016) Homeobox transcription factors and the regulation of meristem development and maintenance. In: Gonzalez DH (ed) Plant transcription factors: evolutionary, structural and functional aspects. Academic Press, London, pp 215–228

[CR40] Veit B, Vollbrecht E, Mathern J, Hake S (1990) A tandem duplication causes the Kn1-O allele of *Knotted*, a dominant morphological mutant of maize. Genetics 125:623–631. 10.1093/genetics/125.3.623 2165968 10.1093/genetics/125.3.623 PMC1204088

[CR41] Verbeke JA (1992) Fusion events during floral morphogenesis. Annu Rev Plant Physiol Plant Mol Biol 43:583–598

[CR42] Vita RS, Menezes NL, Pellegrini MO, Melo-de-Pinna GF (2019) A new interpretation on vascular architecture of the cauline system in Commelinaceae (Commelinales). PLoS ONE 14:e0218383. 10.1371/journal.pone.0218383 31220117 10.1371/journal.pone.0218383 PMC6586312

[CR43] Wang H, Jones B, Li Z, Frasse P, Delalande C, Regad F, Chaabouni S, Latché A, Pech JC, Bouzayen M (2005) The tomato Aux/IAA transcription factor IAA9 is involved in fruit development and leaf morphogenesis. Plant Cell 17:2676–2692 16126837 10.1105/tpc.105.033415 PMC1242265

[CR44] Woerlen N, Allam G, Popescu A, Corrigan L, Pautot V, Hepworth SR (2017) Repression of BLADE-ON-PETIOLE genes by KNOX homeodomain protein BREVIPEDICELLUS is essential for differentiation of secondary xylem in *Arabidopsis* root. Planta 245:1079–1090. 10.1007/s00425-017-2663-2 28204875 10.1007/s00425-017-2663-2

