# Supplementary material for: Ontogeny of postgenital leaf sheath fusion in Commelina erecta L. (Commelinaceae)
Source: Planta. 2026 May 30;264(1):8. doi: 10.1007/s00425-026-05029-4 (PMC13222315; doi:10.1007/s00425-026-05029-4)

```
AtKNAT1  MEEYQ-HDNSTTPQRVSFLYSPISSSNKNDNTSDTNNNNNNNNSSNYGPGYNNTNNNNHHHQHMLFPHMS
AaKN1    MEPFS--------HLGGS--SSSSRATAFMYLPSSTSTPTTQQPPPIAFY--------------------
CpKN1    MEEYNNHVNENSNPRGNFLYPSGVVGGNSSSGNFGRSSNPTAHMGLNSFH-LQQGTDCFQTDHSHPIVKT
TlKN1    MEEIS-HQFGSNNPWGSLMYTFSSPATTTTNTTTASSSTAVVAPPPPPPP---------LGVTPLGFHHN
BpKNOX3  MEDITAHHFG----LGASGHGHG-------HLPWSSSSLSAVVAPPPQQQQQHQ---GYLAPSPLSLNTA
PhKN1    MEEIT-HHFG----VGASGHGHGQHH-HHHHHPWGSSLSAVVAPPPQPPP---------RAGLPLTLNTA
ZmKN1    ----------------------------------------------------------------------
TaKN1    MEEIG-HHFG----LGA--TAHGQHH---SQLPWGSSPLSAVIAPPPQQQQQQQQSAGYLAHSPLSLNTA
OsOSH1   ------------MCRGGL----------------------------------------------------
OsKN1    MEEIS-HHFG---VVGASGVHGGHQH-QHHHHPWGSSLSAIVAPPPPPQLQQQQTQAGGMAHTPLTLNTA
ZoKN1    MEEFS--------QLGGSLWGGIMCSAA--SKPATDHAATVAAAPPPPTS--------------GGTAHA
MaKN1    MEEFS--------QLGGNLWGGLMCSTSP-AAKSSGDTGAVAMVAPPSGG-------------------A
CiKN1    MEEFS--------QLGGNLWGGLMCSTAPAAAKTTADAATVAMVAPPPSG--------------------
CeKN1    ----------------------------------------------------------------------

AtKNAT1  SLLPQTTENCFRSDHDQPNNNNNPSVKSEASSSRINHYSMLMRAIHNTQEANNNNNDNVSDVEAMKAKII
AaKN1    -------------N----------PNPNQLPPKSEPAAAASSSQI------PYARRLVSTDDETIKAKIV
CpKN1    EASSSHHQLHKFHH----------PLLRGGGGGQPPPLTTCRPDQ-----------HGLLDVEAIKAKIL
TlKN1    ------------------------------------------KEA-------SSSQIPLGEVESIKAKIV
BpKNOX3  APSH--------GN----------PVLQLAN-GSLLDACAKAKEP------------YAADVEAIKAKII
PhKN1    ATVNSGAGGN--------------PVLQLANGGSLLDACIKAKEP-------SSSSLYAGDVEAIKAKII
ZmKN1    ----------------------------------------------------------------------
TaKN1    PPSGSHGGGSGCSN----------PVLQLAN-GSLLEACAKAAKE-------PSSSSYAADVEAIKAKII
OsOSH1   ----------------------------------------------------------------------
OsKN1    AAA--------VGN----------PVLQLAN-GSLLDACGKAKEA-------SASASYAADVEAIKAKII
ZoKN1    SVFDKAGGGCGIAA----------PTLLAGHQNSAAALLFQGKHEGGVGGASSSHFPPASDIDAIKAKIV
MaKN1    SVFDK-GHGC-IPN----------PLLL-GSGGNQNS-HLLAKHE----GASSSHFPP-CDVDAIKAKII
CiKN1    -------HGCNIPN----------PTTLLNNQNTLN---LQGKHE----GASSSHFPPGADVAAIKSKII
CeKN1    ----------------------------------------------------------------------

AtKNAT1  AHPHYSTLLQAYLDCQKIGAPPDVVDRITAARQDFEARQQRSTPSV--SASSRDPELDQFMEAYCDMLVK
AaKN1    SHPQYSALLGAYMDCQKVGAPPELAARLSVIAREIEAQQQAAAASCRRDASSTDPELDQFMEAYCNMLVK
CpKN1    AHPQYSSLLEAYMECQKVGAPPQVVERLVAARHEFETRQRSSMVSG---ETIKDPELDQFMEAYYDMLVK
TlKN1    SHPYYSSLLAAYLDCQKVGAPPEVVARLSSVAQDLEARQRAALSCL---DAPTEPELDQFMEAYHEMLVK
BpKNOX3  SHPIYPSLLAAYLDCLKVGAPPEVSERMSAVARDLELRQRAGLGGL---AAATEPELDQFMEAYSEMLVK
PhKN1    SHPHYYSLLAAYLECQKVGAPPEVSARLTAMAQELEARQRTALGGL---GAATEPELDQFMEAYHEMLVK
ZmKN1    ------------------------------------------------------------MEAYHEMLVK
TaKN1    SHPHYSSLLAAYLDCQKVGAPPEVLARLTAVAQDLELRQRTALGSL---GTATEPELDQFMEAYHEMLVK
OsOSH1   ----------------QVGAPPEVAARLTAVAQDLELRQRTALGVL---GAATEPELDQFMEAYHEMLVK
OsKN1    SHPHYSSLLAAYLDCQKVGAPPEVAARLTAVAQDLELRQRTALGVL---GAATEPELDQFMEAYHEMLVK
ZoKN1    SHPQYSNLLTAYIDCQKVGAPPEVVDRLSAVAQELEMRQRASLICR---DTHTDPELDQFMEAYHEMLVK
MaKN1    SHPQYSSLLAAYIDCQKVGAPPEVVDRLSAVAQELEMRQRASLICR---DAPTDPELDQFMEAYHEMLVK
CiKN1    SHPQYSSLLAAYIDCQKVGAPPEVVDRLSAVAQELEMRQRASLICQ---DAPTDPELDQFMEAYHEMLVK
CeKN1    --------------------------------------------------------------------VK
```

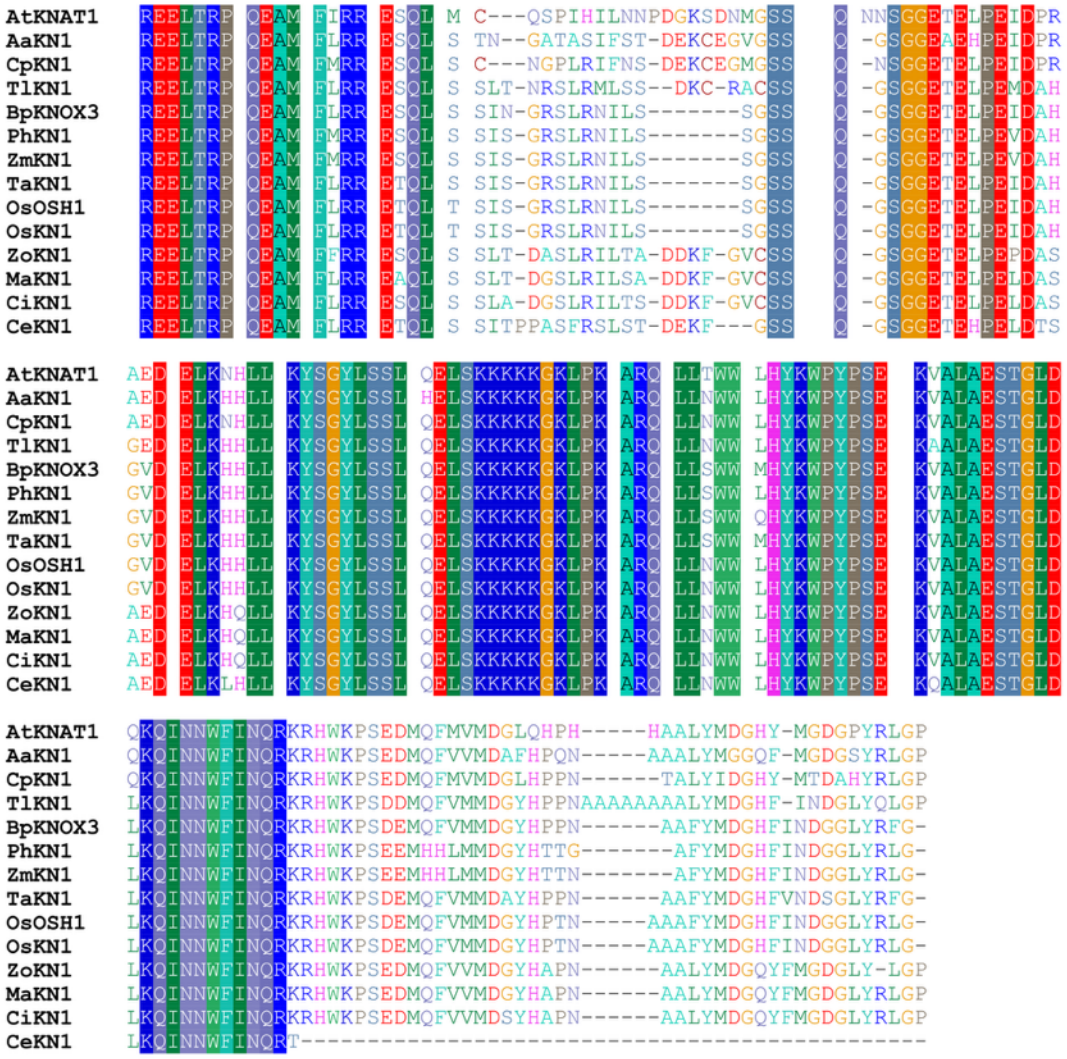

Supplement: Supplementary file 1 — Supplementary file1 (PDF 1367 KB) [file 425_2026_5029_MOESM1_ESM.pdf]
